# Anticoagulant use in older persons at risk for falls: therapeutic dilemmas—a clinical review

**DOI:** 10.1007/s41999-023-00811-z

**Published:** 2023-07-01

**Authors:** Anneka Mitchell, Yasmin Elmasry, Eveline van Poelgeest, Tomas J. Welsh

**Affiliations:** 1 Research Institute for the Care of Older People (RICE), Bath, UK; 2 Pharmacy Department, University Hospitals Plymouth NHS Trust, Plymouth, UK; 3 Amsterdam University Medical Centers, Amsterdam, The Netherlands; 4 Bristol Medical School, University of Bristol, Bristol, UK; 5 Royal United Hospitals Bath NHS Foundation Trust, Bath, UK; 6 Life Sciences Department, University of Bath, Bath, UK

**Keywords:** Anticoagulants, Falls, Deprescribing, Geriatric, Atrial fibrillation, Venous thrombosis

## Abstract

**Purpose:**

The aim of this clinical narrative review was to summarise the existing knowledge on the use of anticoagulants and potential adverse events in older people at risk of falls with a history of atrial fibrillation or venous thromboembolism. The review also offers practical steps prescribers can take when (de-)prescribing anticoagulants to maximise safety.

**Methods:**

Literature searches were conducted using PubMed, Embase and Scopus. Additional articles were identified by searching reference lists.

**Results:**

Anticoagulants are often underused in older people due to concerns about the risk of falls and intracranial haemorrhage. However, evidence suggests that the absolute risk is low and outweighed by the reduction in stroke risk. DOACs are now recommended first line for most patients due to their favourable safety profile. Off-label dose reduction of DOACs is not recommended due to reduced efficacy with limited reduction in bleeding risk. Medication review and falls prevention strategies should be implemented before prescribing anticoagulation. Deprescribing should be considered in severe frailty, limited life expectancy and increased bleeding risk (e.g., cerebral microbleeds).

**Conclusion:**

When considering whether to (de-)prescribe anticoagulants, it is important to consider the risks associated with stopping therapy in addition to potential adverse events. Shared decision-making with the patient and their carers is crucial as patient and prescriber views often differ.

## Key summary points

**Aim:**

To summarise the existing knowledge on the benefits of anticoagulants, and adverse events associated with falling whilst taking these medications, to assist clinicians in decisions on safe prescribing and deprescribing of anticoagulants.

**Findings:**

Anticoagulants may increase the risk of intracranial haemorrhage associated with falling, but the absolute risk is relatively low compared to the risk of ischaemic stroke and venous thromboembolism. Addressing modifiable risk factors for falls and bleeding can make anticoagulant therapy safer.

**Message:**

Clinicians often cite falls risk as a reason not to prescribe anticoagulant therapy, but this increases the patient’s risk of stroke/venous thromboembolism.

## Introduction

Falls occur frequently in older people and the risk of falls increases with age, half of people aged over 80 years will fall at least once a year [1]. Falls are a major concern as 5–10% will cause serious injury [2], they increase mortality [3], and can reduce confidence and independence [1]. Falls can enhance functional and cognitive decline and increase reliance on both formal and informal care [4]. Multiple risk factors for falls have been identified, including sociodemographic factors, mobility impairment, gait or balance difficulties, comorbidities and medications [2].

Atrial fibrillation (AF) is common in older people, and the incidence increases substantially with advancing age. The incidence of AF has been estimated as 20.7 per 1000 person-years in those aged 80–84 years compared with 1.1 per 1000 person-years in the 55–59 year age group [5]. Atrial arrhythmias have been identified as two of the most common cardiovascular causes of falls [6]. Syncope and falls may also be a sign of AF in those yet to be diagnosed [7]. In patients who are severely frail, the rate of falls may be up to eight times higher in those with AF than those without [8]. Furthermore, AF significantly increases the risk of stroke. Strokes associated with AF are often more severe and are associated with a higher rate of disability and mortality than those occurring in people without AF. Effective stroke prevention is therefore a key component of AF management [9, 10].

Anticoagulation has been shown to significantly reduce the risk of stroke in AF, particularly in older people who are at greatest risk [11]. However, anticoagulation has historically been underused in this group [12]. One of the major reasons cited by physicians for withholding anticoagulation is risk of falls [13–15]. Despite falls risk being cited in a number of research studies as a barrier to prescribing of anticoagulants, it is often not discussed with patients. Therefore, patients are not adequately involved, and may feel detached from the decision-making process [16–18]. Studies evaluating patient preferences for anticoagulation have demonstrated that their perception of risk and benefit often differ to those of prescribers, and that patients value stroke prevention more highly than other factors such as bleeding risk, but risk tolerance is highly variable [19, 20]. Patients may also not receive early anticoagulation because AF can be asymptomatic in 50–87% of cases, and only identified later either incidentally as part of routine care or following a stroke [21]. There have been a number of initiatives to implement routine screening for AF, particularly for older people, but evidence to support routine screening is limited, and results of ongoing randomised controlled trials are awaited [22].

The UK National Institute for Health Care Excellence (NICE) updated their AF guidelines in 2021 to specifically state that anticoagulation should not be withheld solely due to a person’s age or risk of falls [23]. The European Society of Cardiology (ESC) guidelines also advise that “a history of falls is not an independent predictor of bleeding on an oral anticoagulant” and an increased risk of falls does not outweigh the benefits of anticoagulation in older patients [21]. However, previous work by our group has shown that older patients with AF and a history of falls in the UK were 17% less likely to receive anticoagulation than those with no prior falls, and previous fracture reduced anticoagulant prescribing by 12% [24]. Similar findings have also been reported for older people with dementia who fall [25] and a substantial proportion of patients included in the ORBIT-AF registry discontinued warfarin due to falls [26].

Anticoagulation is less likely to be avoided in older patients with a confirmed venous thromboembolism (VTE) in the initial phase; however, weighing up the risk of VTE recurrence and the risk of bleeding when considering extended anticoagulant treatment can be difficult in older patients who are at increased risk of both.

The aim of this narrative review is to call attention to the risks of both prescribing and not prescribing anticoagulants to older people at increased risk of falls, and to assist prescribers in appropriate use of these agents in the two most common indications, VTE and AF.

This review was informed by a literature search conducted in August 2022 and updated in January 2023 in Pubmed, Embase and Scopus. Searches were conducted using a combination of keyword, free text, MeSH and Emtree headings for the three key topics: older adults, anticoagulants and falls. Reference lists were searched for further relevant literature.

## Medication review and reconciliation

### Match anticoagulant use to an appropriate indication

Medication review is a key component of multifactorial interventions to reduce falls risk and harm from falls [27]. This review should include the patient/carer and review all medications including those purchased over the counter and herbal or alternative medicines [28]. Establishing the indication for anticoagulation is important, the two most common indications are AF and VTE, and these are the indications that this review will focus on. Besides AF and VTE, there are a number of other indications for anticoagulation, including venous thrombosis prophylaxis (which may be a prolonged course following some operations e.g., hip replacement or if mobility is likely to be reduced for a substantial period of time) [29]; life-long following implantation of a mechanical prosthetic heart valve [30]; for up to 3 months after surgical implantation of a bioprosthetic heart valve [30].

Anticoagulation is effective in reducing the risk of stroke in people with AF, and is usually required long term (unless the patient has a successful ablation procedure, requiring anticoagulation therapy for only 8 weeks after ablation). One meta-analysis found that the number needed to treat (NNT) with warfarin for 1 year to prevent one stroke was 37 for primary prevention (i.e., those with no history of stroke or TIA) and 12 for secondary prevention [31]. However, the benefit of anticoagulation is greatest in those with a moderate to high risk of stroke and may be less pronounced in those with a low risk [31]. The CHA_2_DS_2_-VASc score is recommended to assess stroke risk and guide whether to treat with anticoagulation [21, 23]. Being aged ≥ 75 years with no other risk factors for stroke, gives a CHA_2_DS_2_-VASc score of 2 and guidelines recommend anticoagulation be considered in men with a score ≥ 1 or women with a score ≥ 2 [21] or ≥ 2 in both sexes [23] meaning that all older patients should be considered for treatment. Stroke risk increases further with certain comorbidities including congestive heart failure, hypertension, previous stroke or thromboembolism and vascular disease, which are also included in the score. The risk of stroke should then be weighed against the risk of bleeding, with guidelines recommending different scores. NICE recommends the ORBIT score as they believe it better discriminates between those at high and low risk of bleeding [23, 32], whereas the ESC recommend the HAS-BLED score which has been used to assess major bleeding (defined as fatal bleeding; bleeding into a critical area or organ; a bleed causing a fall of ≥ 20 g L^−1^ or leading to transfusion of two or more units of whole blood or red cells [33]) risk in AF for a number of years [21, 34]. HAS-BLED has become outdated now that warfarin and other vitamin K antagonists (VKAs) are less commonly used (it includes labile international normalised ratio (INR) as a variable); ORBIT can be used for both DOACs and VKAs. There is debate as to which score better identifies those patients who are truly high risk for bleeds [32, 34–37]. Whichever score is used, the key aim is to identify and address potentially modifiable risk factors for bleeding, such as (uncontrolled) hypertension, other medication and alcohol use, rather than suggesting absolute contraindication to anticoagulant therapy.

Following the first episode of a proximal deep vein thromboembolism (DVT) or pulmonary embolism (PE), anticoagulation is recommended for all patients for a minimum of 3 months and up to 6 months for those with active cancer [38, 39]. Where a major transient or reversible risk factor for the venous thromboembolism can be identified and is no longer present after 3–6 months then the anticoagulant should be discontinued [38, 39]. Long-term anticoagulation is actively recommended for those with recurrent VTE that is not related to a major transient or reversible risk factor and those with antiphospholipid syndrome [39]. For patients with no identifiable risk factor, those with a persistent risk factor, and those with first VTE and only minor transient risk factor long-term anticoagulation should be considered, but the risk of recurrence of VTE must be balanced against the risk of bleeding [38, 39]. The HAS-BLED score can be used to guide decision-making, with consideration given to stopping anticoagulation in those with a score ≥ 4. However, this should only be considered after addressing modifiable risk factors [38].

### Choice of anticoagulant

Historically, VKAs such as warfarin were the only oral anticoagulants available, but from 2008, a new class of oral anticoagulants was licensed, the DOACs. These DOACs offered important advantages over the VKAs as they have fixed dosing regimens, fewer drug–drug and drug–food interactions, and do not require regular blood tests [40].

Multiple randomised controlled trials have shown DOACs to be as effective as warfarin for stroke prevention in older people AF [41–43], they were also associated with a significantly lower risk of intracranial haemorrhage [41–43], which is an important consideration when prescribing these medications for people at risk of falls. Observational registry data also showed that for older people (aged ≥ 75 years), the net clinical benefit, incorporating both stroke risk reduction and bleeding risk, was greater with DOACs than warfarin [44]. Meta-analyses of both randomised controlled trial and observational data further confirmed these findings, showing that DOACs were as effective as [45] or superior to VKAs [46] for stroke prevention. DOACs were also associated with less [46] or similar [45] rates of major bleeding and a significant reduction in the risk of intracranial haemorrhage [45].

The pivotal trials for the use of DOACs to prevent VTE recurrence showed them to be non-inferior to conventional therapy (subcutaneous enoxaparin followed by warfarin), with lower (apixaban and edoxaban) [47, 48] or similar (dabigatran and rivaroxaban) [49–52] rates of major bleeding. A meta-analysis of phase three trials found similar rates of recurrent VTE, fatal PE and overall mortality with DOACs and conventional therapy [53]. Risk of major and non-major bleeding, intracranial haemorrhage and fatal haemorrhage were significantly lower in the DOAC group overall [53]. The benefits of DOACs may be even greater for older people, a meta-analysis using data for patients aged ≥ 75 years found that DOACs reduced the risk of VTE recurrence compared with warfarin and were associated with less major bleeding [54].

To date, there have been no head-to-head trials comparing the DOACs to each other. A large network meta-analysis compared the efficacy, safety and cost-effectiveness of the DOACs to one another and found that in AF, apixaban was ranked best for most outcomes. Dabigatran was associated with a lower risk of ischaemic stroke and systemic embolism than edoxaban and rivaroxaban, there was no significant difference between dabigatran and apixaban. The risk of major bleeding was lower with apixaban than either dabigatran or rivaroxaban, and intracranial bleeding was significantly lower with apixaban than all other DOACs [55]. Because this network meta-analysis did not stratify the analyses by age, it is unclear how well the results would extrapolate to older people.

Anticoagulation with warfarin has been shown to significantly reduce the risk of ischaemic stroke in patients with dementia [56, 57] without increasing the risk of intracranial haemorrhage [57]. Clinically relevant bleeding appeared to be increased in patients with dementia treated with warfarin compared to those treated with warfarin who did not have dementia. However, due to the small sample size, this was associated with a wide confidence interval [56]. There is a paucity of evidence on the safety of DOACs for patients with dementia. One study found that DOACs were associated with a similar risk of ischaemic stroke and a reduced risk of intracranial haemorrhage, but they were associated with an increased risk of gastrointestinal bleeding and mortality compared with warfarin in people with dementia [58].

Recent (inter)national guidelines now recommend DOACs first line for anticoagulation in both AF and VTE [21, 23, 38, 39], unless there are contraindications such as severe renal impairment, for AF patients with mechanical prosthetic heart valves or moderate to severe mitral stenosis, or VTE patients with antiphospholipid syndrome, where vitamin K antagonists are still the preferred choice [2, 38]. Aspirin is sometimes prescribed as an alternative to anticoagulation as prescribers feel it is safer [59]; however, antiplatelet agents are no longer recommended for stroke prevention in AF as they are substantially less effective for stroke than anticoagulation, whilst being associated with a similar risk of major bleeding [11, 60].

For patients with AF on VKAs in whom a DOAC would be suitable, UK guidance recommends that the option of proactive switching is discussed at a routine appointment, particularly in patients with labile INR and low time in therapeutic range [23]. Switching is only recommended by European guidance where patients are admitted with acute ischaemic stroke despite taking an anticoagulant. In this case, if a VKA is prescribed, it is recommended to optimise time in therapeutic range or to switch to a DOAC. If already taking a DOAC, then adherence and dose should be checked [21]. The evidence to support switching from VKAs to DOACs is limited. A recent meta-analysis of observational studies found that the risk of stroke, myocardial infarction and gastrointestinal bleeding was increased, but the risk of intracranial bleeding was decreased when switching from a VKA to dabigatran [61], the risk of gastrointestinal bleeding was also increased when switching from a VKA to rivaroxaban [61]. However, the reason for switching may confound observational studies and the results of the multicentre, randomised controlled trial (FRAIL-AF) are awaited to provide a more definitive answer [62].

Patients with cancer presenting with VTE are at particularly high risk of VTE recurrence whilst also being at an increased risk of bleeding due to both the cancer and the anti-cancer drug treatments. Guidance from the ESC and American Society of Clinical Oncology (ASCO) recommends that low molecular weight heparins (LMWH) are used first line for the first 6 months of treatment in preference to VKAs [39, 63]. Edoxaban or rivaroxaban can be considered as alternative options provided the patient does not have gastrointestinal cancer [39, 63]. The 2022 International Clinical Practice Guidelines for the treatment and prophylaxis of venous thromboembolism in patients with cancer recommend that rivaroxaban, apixaban or edoxaban can be used for patients without a high risk of gastrointestinal or genitourinary bleeding [64]. All three DOACs have been shown to be non-inferior to LMWH in preventing recurrent VTE [65–68]. Edoxaban and rivaroxaban were associated with an increased risk of major bleeding especially in those with gastrointestinal cancer [65, 66], a finding not demonstrated with apixaban [67, 68]. Patients with AF and cancer are also at increased risk of bleeding, but guidelines still recommend that DOACs are used first-line unless the patient has gastrointestinal cancer, or there are significant drug interactions between the DOAC and the anti-cancer medication [69].

### Anticoagulant dosage

**Fig. 1:**
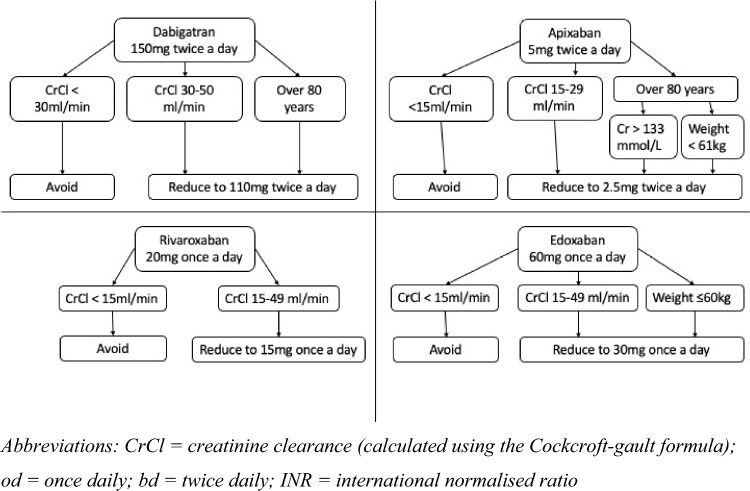
Summary of oral anticoagulant doses for stroke prevention in atrial fibrillation

The recommended dosing for each of the DOACs recommended for stroke prevention in AF is shown in Fig. 1 [70–73]. Warfarin should be dosed based on the INR, aiming for a range of 2–3.

Under dosing of DOACs in older patients who do not meet the criteria listed in Fig. 1 is relatively common, occurring in 20–39% of patients prescribed DOACs for stroke prevention in AF [74, 75]. In the ORBIT-AF registry, 57% of patients were prescribed a low-dose DOAC that had their dose reduced inappropriately [76]. Advancing age, higher CHA_2_DS_2_-VASc score and history of renal impairment have been associated with off-label dose reduction [74]; however, this is not consistent between all studies and may also be attributable to confusion between the differing dose reduction criteria between DOACs [76]. In clinical practice, we often encounter off-label dose reductions in patients who are at risk of or have experienced one or more falls, or frail patients who are considered to be at particularly high risk of bleeding. Off-label dose reduction has been associated with higher rates of thromboembolism and death [75, 76], and no change [48] or an increase in major bleeding compared with prescription of the licensed dose [75]; therefore, it cannot be recommended.

**Fig. 2:**
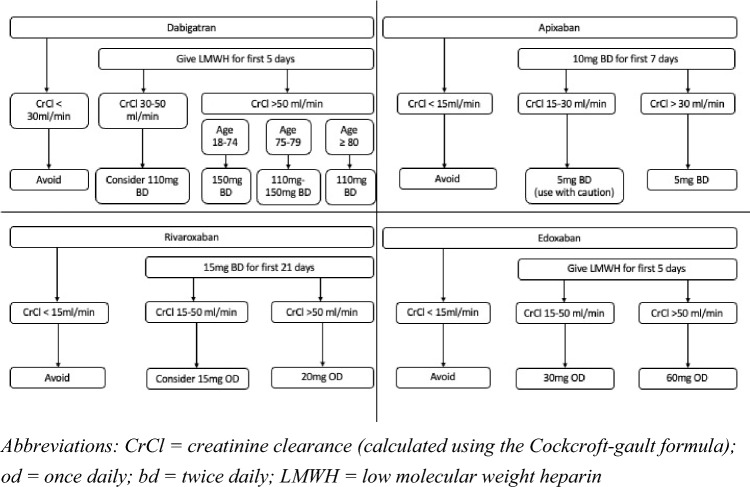
Summary of oral anticoagulant doses for stroke prevention in venous thromboembolism

Figure 2 summarises the dosing schedules for each anticoagulant for prevention of recurrence of VTE [70–73]. It should be noted that patients must receive 5 days of treatment with a parenteral anticoagulant before commencing dabigatran or edoxaban. This is not required for rivaroxaban or apixaban. Parenteral treatment should be continued alongside warfarin until there are two consecutive INR readings between 2 and 3.

After the initial 3–6 month dosing period, the decision on whether extended anticoagulant treatment is indicated must be made. The criteria for extended treatment are discussed in the previous sections. The doses for extended treatment differ between the DOACs. The dose of rivaroxaban can be reduced to 10 mg daily, although a higher dose of 20 mg should be considered in patients with complex co-morbidities and high risk of recurrent VTE [71]. For apixaban, the dose is reduced to 2.5 mg twice daily regardless of risk [72]. For dabigatran and edoxaban, the dosing regimens for extended treatment are the same as for the initial phase as shown in Fig. 2 [70, 73].

Rivaroxaban, apixaban and dabigatran have all been shown to reduce the risk of recurrent VTE with no significant increase in the risk of major bleeding [77–79]. A recent network meta-analysis confirmed this, showing that all DOACs and vitamin K antagonists were associated with a significantly lower risk of recurrent VTE with extended treatment; however, only vitamin K antagonists were associated with a higher risk of major bleeding than aspirin or placebo [80]. However, the evidence for extended VTE treatment and anticoagulant dosing has largely been derived from randomised controlled trials which under-represented older people, only 15% of those included in the apixaban trial were aged ≥ 75 years [79], and the average ages of participants in the rivaroxaban and dabigatran trials were 58 and 56 years, respectively [77, 78]. None reported outcomes for the subgroup of older people and the risk/benefit ratio may therefore differ in the older population who are known to be at higher risk for VTE but also bleeding.

It is well known that pharmacogenetics plays a substantial role in patients’ response to warfarin, with variations in CYP2CP and vitamin K epoxide reductase complex subunit 1 gene VKORC1 contributing to the wide inter-person variability [81]. Warfarin dosing algorithms that incorporate genetic information via point of care testing have been shown to improve the time in therapeutic range and reduce the incidence of excessive anticoagulation [82, 83]. Elucidating the effects of pharmacogenomics on the DOACs is still in its infancy. There is some evidence that variations in CES1 and ABCB1 may be associated with differences in peak and trough concentrations of dabigatran and also the risk of minor bleeding [84]; however, there is no strong evidence at present that pharmacogenomics would improve the safety or efficacy of any of the other DOACs [84].

## Risk of falls in patients on anticoagulants

Anticoagulants are not commonly associated with increasing the risk of falls; however, there are differences in the adverse effects between the different medications that may influence falls risk. Rivaroxaban and edoxaban have dizziness listed as a common side effect [71, 73]. Hypotension is listed as a common side effect of rivaroxaban and an uncommon side effect of apixaban [71, 72]. Syncope and decreased strength and energy are also listed as common side effects of rivaroxaban [71].

The mechanism of these adverse effects is not yet known; however, animal models suggest that both apixaban and rivaroxaban may have vasodilatory effects which if extrapolated to humans could explain the dizziness, hypotension and syncope experienced by some patients [85, 86].

## Risks associated with anticoagulant use in persons falling

Prescribers often cite increased falls risk as a reason not to prescribe anticoagulation to older people [13–15] due to the adverse events potentially associated with being anticoagulated at the time of a fall. The following paragraphs summarise the evidence for adverse outcomes following a fall and aim to put this into context with the risk of harm from omitting anticoagulant therapy.

### Fractures

Vitamin K is involved in bone metabolism, so it has been hypothesised that vitamin K antagonists (VKAs) would increase the risk of fracture. Various studies have evaluated fracture risk with long-term VKA therapy but have found inconsistent results with some studies finding an increased risk [87–89] and others finding no difference [90, 91]. A meta-analysis found that VKAs were not associated with an increase in fracture risk when compared to either controls or DOAC users [92]. More recently, a large observational study found that DOAC use was associated with a reduced risk of fracture compared with warfarin, there was no difference in fracture risk when the DOACs were compared with each other [93].

### Intracranial haemorrhage

Intracranial haemorrhage is one of the most feared complications of anticoagulant therapy as it is a serious condition associated with a substantial increase in mortality [94]. The risk of both traumatic and non-traumatic intracranial haemorrhage (ICH) is substantially increased in people at high risk of falls. One study found the incidence of traumatic and non-traumatic intracranial haemorrhage more than doubled in AF patients defined as having a high falls risk when compared with lower risk patients regardless of whether they were taking an anticoagulant or not [95].

Several studies have assessed the risk of traumatic ICH (tICH) following ground-level falls in patients taking anticoagulants. However, they are often small and conducted in single trauma centres or emergency departments [96–98]. These studies found no increase in the risk of tICH compared with people not on anticoagulant therapy or those taking antiplatelet agents [96, 98].

The risk of ICH has commonly been cited as a reason to avoid warfarin therapy in people at risk of falls; however, evidence is conflicting as to whether warfarin increases the risk of ICH occurrence [95, 99, 100]. There is also debate on whether warfarin increases the risk of mortality from ICH [95, 97, 100, 101] or not [99], although the majority of studies suggest it does. All four of the pivotal DOAC randomised controlled trials in patients with AF demonstrated that DOACs were associated with significantly lower risk of ICH than warfarin [102–105], a result also seen in a meta-analysis of observational studies of older people with AF [45], so these agents may be preferable to warfarin in people at risk of falls. A retrospective single-centre study of 1453 elderly patients admitted with tICH found comparable rates of injury severity score, mortality and rehabilitation between patients admitted on DOACs prior to injury compared with to those who were not on anticoagulants [97]. Conversely, warfarin use increased the risk of in-hospital mortality or discharge to hospice care [97]. Interestingly, patients on DOACs had better outcomes despite being less likely to receive reversal agents (idarucizumab and andexanet alfa) and being more likely to require surgical intervention compared to patients on a VKA [97].

Subgroup analyses of the apixaban (ARISTOTLE) and edoxaban (ENGAGE AF-TIMI 48) trials found that patients with a history of falling had higher rates of ICH and death than those who had never fallen [106, 107], but there was no difference between apixaban and warfarin on these outcomes [106]. The absolute risk reduction of ICH in patients treated with edoxaban compared with warfarin was greater in those assessed as being at high risk of falls [107]. A recent meta-analysis that included subgroup analyses of randomised controlled trials and retrospective cohort studies comparing outcomes in patient at high of falls suggested that the risk of ICH was approximately 50% lower with DOACs than VKA [108].

A large observational study using Medicare data from the USA found that DOAC use was associated with a 43% reduction in the risk of ICH compared with warfarin in people at high risk of falls (predicted two-year fall risk of ≥ 15%) [109].

Debate is ongoing regarding the utility and cost-effectiveness of performing a CT scan on all patients who sustain a mild head injury who are taking an anticoagulant. UK NICE guidance currently recommends that anyone taking an anticoagulant who suffers a head injury should have a CT head scan within 8 h of the injury [110], but this has been downgraded in the draft guideline, currently under consultation, to just considering a CT head scan where there are no other indications to do one [111]. Patients presenting to hospital alert and with no associated symptoms are at low risk of adverse outcomes [112, 113], so it can be argued that CT head scans may be of little value in this patient group and that an individualised approach is required as opposed to routine scanning. The risk of radiographic head injury in hospital in-patients who fall is likely to be lower still than those presenting to the emergency department following a fall in the community, but patients taking anticoagulants frequently undergo CT head scans [114]. There is a need for more detailed guidance on when people taking anticoagulants require a CT head scan, but further research is needed [114].

Cerebral microbleeds are known to significantly increase the risk of ICH; however, they may also increase the risk of ischaemic stroke [115]. Cerebral microbleeds may be identified on magnetic resonance imaging post stroke, but in older adults, they may also be identified incidentally during cognitive screening which presents a therapeutic dilemma on whether to start or continue anticoagulation. Studies have suggested that increasing numbers of cerebral microbleeds may confer an increased risk of ICH [116, 117], with one systematic review suggesting that the presence of ≥ 5 cerebral microbleeds could identify patients with AF who are at high risk of ICH from anticoagulation [116]. The location of the microbleeds may also an important consideration, lobar microbleeds suggestive of cerebral amyloid angiopathy have been associated with an increased risk of ICH but not ischaemic stroke, whereas microbleeds in other locations have been associated with both an increased risk of ischaemic stroke and ICH [118]. However, pooled analyses have refuted this, suggesting that the location or distribution of the microbleeds does not influence the risk of ischaemic stroke or ICH [119]. When prescribing anticoagulation for patients who fall, the increased risk of ICH associated with both falls and cerebral microbleeds must be considered and carefully weighed against the risk of stroke. For patients with cerebral microbleeds, no history of TIA or stroke and a low overall risk, the risk of ICH outweighs the risk of ischaemic stroke. However, for patients with a history of ischaemic stroke or TIA, it is likely that the reduction in risk of recurrent stroke will outweigh the increased risk of ICH as shown by Wilson and colleagues in a very large pooled analysis of cohort data [119].

### Morbidity and mortality

There is debate about whether anticoagulant therapy effects the risk of mortality in older people who fall. Data from the large National Trauma Databank suggest that having a fall whilst taking an anticoagulant is associated with an increase in the likelihood of death of 180% compared to falling whilst not taking an anticoagulant [120]. It should be noted, however, that the injuries differed substantially between the group taking anticoagulants and those who were not so anticoagulant therapy may not be the sole reason for this increase [120]. Antithrombotic therapy (including both anticoagulants and antiplatelets) has not been associated with an increase in overall or in-hospital mortality following a fall and traumatic brain injury. However, people taking pre-injury antithrombotic therapy may be more likely to be discharged to a nursing home or rehabilitation facility than those not taking these medications at the time of the fall [99]. A systematic review comparing pre-injury DOAC use with warfarin in older patients with a traumatic brain injury found no difference in mortality, hospital or intensive care length of stay, or need for surgical intervention [121].

### Risk-to-benefit ratio

A number of studies have sought to estimate the point at which the risks associated with falling whilst taking an anticoagulant outweigh the beneficial effect of stroke risk in AF. An older study that is referenced in most national and international guidelines estimated that someone would have to fall almost daily (295 times in a year) for the risk of intracranial bleeding with warfarin to outweigh the benefits [122]. A similar study gave a more conservative estimate for warfarin of 35 falls per year as they included a broader range of risks. They also estimated that someone would need to fall 45 times a year for rivaroxaban or 458 times a year for apixaban for the risk to outweigh the benefit [123]. These studies were both based on Markov models, so the calculations are reliant on the underlying assumptions of baseline and outcome risks extracted from the literature and applied to a theoretical cohort.

For patients that have experienced an intracranial bleed whilst taking an anticoagulant, the risk-to-benefit ratio is likely to shift and requires consideration of both the risk of recurrent intracranial bleeding and the ongoing thromboembolic risk as both are associated with considerable morbidity and mortality.

Following a traumatic intracranial haemorrhage, it is generally accepted that anticoagulation should be restarted as it reduces ischaemic events and mortality without increasing the risk of recurrent intracranial haemorrhage [124–126]. However, the time to anticoagulant resumption is still a subject of debate with a global survey of clinicians finding that they opted to restart anywhere between 1 week and 3 months post-haemorrhage [125]. There is some evidence to suggest that anticoagulation should restart sooner. A systematic review found that recurrent haemorrhagic complications were most common in the first 3 days following the index event, whereas thromboembolic complications occurred later. When time to restarting anticoagulation was evaluated, they found that longer delays (5–7 days as opposed to 3 days) significantly increased the risk of thromboembolism. However, the risk of recurrent haemorrhage was attenuated with longer delays [127]. The current evidence evaluating when to restart anticoagulation looks solely at warfarin treatment and is largely based on non-randomised studies. The ongoing Restart TICrH randomised trial will provide further clarity on the optimal time to restart anticoagulation with DOACs in patients with traumatic intracranial haemorrhage [128].

## Drug–drug interactions

Several different drug–drug interactions need to be considered when evaluating the risks associated with anticoagulant therapy. Warfarin has a narrow therapeutic index and has many drug–drug and drug–food interactions that may increase the risk of bleeding or reduce the effectiveness of the medication [129]. Warfarin therefore requires regular monitoring of the international normalised ratio (INR), particularly if concomitant therapy or diet changes.

The DOACs have fewer drug–drug interactions, but there are some significant interactions that must be considered when prescribing. Dabigatran and edoxaban (to a lesser extent) are substrates for the P-glycoprotein (P-gp) efflux transporter, so medications that inhibit P-gp (e.g., ketoconazole, dronedarone) would be expected to increase the concentrations of dabigatran and edoxaban and increase the risk of bleeding, whereas P-gp inducers (e.g., rifampicin, carbamazepine) may reduce their effectiveness [70, 73]. Rivaroxaban and apixaban are metabolised by both CYP3A4 and P-gp; therefore, strong inhibitors of either pathway may increase the risk of bleeding [71, 72]. The Summary of Product Characteristics (SmPCs) or other reputable source, such as Stockley’s drug interactions, Medscape or UpToDate interaction checker, should be used to guide dose adjustment and contraindications due to these interactions. The 2018 European Heart Rhythm Association Practical Guide on the use of non-vitamin K antagonist oral anticoagulants in patients with atrial fibrillation also provides a useful summary table of the relevant drug–drug interactions which can be used to select the DOAC with the least potential for drug–drug interactions and guide a personalised approach to prescribing [130].

Other drug interactions that are likely to increase the risk of harm from falling while on anticoagulants are those that are also known to increase the risk of bleeding. There are only limited situations in which antiplatelets are recommended to be used alongside anticoagulants, for example following acute coronary syndrome. Unless patients are at particularly high risk from coronary artery disease and have a low risk of bleeding, it is recommended that triple therapy (anticoagulant plus two antiplatelets) is limited to 1 month, double therapy (anticoagulant plus a single antiplatelet) for up to 1 year, then the anticoagulant should be continued alone [131]. Other medications that can increase the risk of bleeding include selective serotonin reuptake inhibitors (SSRIs) and non-steroidal anti-inflammatories (NSAIDs), so the need for these medications should be carefully considered when used in addition to anticoagulation.

## Reducing the risk of anticoagulants by (de)prescribing

### Considerations when deciding whether to prescribe anticoagulants

There are a number of factors that can reduce the risk of bleeding when prescribing anticoagulants to people at risk of falls:

1. Medication review should be undertaken as part of a comprehensive geriatric assessment before commencing anticoagulants and where new medications are being considered for patients already prescribed anticoagulants.
2. Falls risk increasing drugs should be discontinued where clinically appropriate or switched to a lower risk alternative. One example of this in the context of AF, could be switching digoxin to a beta-blocker. A meta-analysis found that digoxin can double the risk of falling, conversely beta-blockers (also used for rate control) may reduce the risk of falls [132]. A large observational study in Denmark found that digoxin monotherapy increased the risk of fall-related injury [133].
3. Interacting, contraindicated or unnecessary medication should be stopped or switched.
4. Fall prevention measures should be implemented including physical training to improve strength, balance and gait; provision of walking aids; environmental hazards should be identified and addressed; footwear optimised; visual impairments evaluated and treated
5. Any decision to prescribe anticoagulation should be driven by patient goals and wishes. The discussion should be informed by the use of risk calculators and facilitated using shared decision making tools. Using shared decision making tools can ensure that patients are fully informed of the risks and benefits of a treatment and also help to ensure that their treatment values are recognised. A number of tools have been created on different platforms to assist shared decision making in the context of anticoagulation for AF, these are summarised and evaluated by Torres Roldan and colleagues [134]. It should be noted that shared decision making tools often present average risks and benefits and additional information may need to be provided to higher risk patients, such as those at risk of falls.
6. Anticoagulant choice should be based on both the evidence base and patient preference. DOACs have a similar efficacy profile but improved safety compared to vitamin K antagonists and are preferred for most patients. To date, evidence suggests that apixaban is associated with the lowest risk of bleeding and is likely to be the preferred option [106, 108, 135]. If a once daily DOAC is required, edoxaban has a favourable safety profile compared with rivaroxaban [107, 135].

### Considerations when deciding to deprescribe anticoagulants

The comprehensive geriatric assessment should be used to guide decisions on deprescribing anticoagulants:

1. End of life care/severe frailty—where a focus on symptom control and a comfort-orientated approach may be more appropriate, consideration to deprescribing anticoagulation should be given. Granziera and colleagues suggest that anticoagulation should be stopped in those with ≤ 6 months life expectancy [136]; however, it should be noted that the evidence to support this recommendation is limited. In-depth chart review for patients hospitalised in the last three months of life showed that de-prescribing of anti-thrombotics is often reactive and in response to bleeding or sudden deterioration [137]. There is a need for clear clinical guidance to help clinicians when making these difficult decisions and to help them explain the relative thrombotic and bleeding risks as patients approach the end of their life.
2. Significant risk factors for major bleeding—this might include problems such as recent peptic ulceration, recent intracranial haemorrhage or known oesophageal varices. A risk calculator such as ORBIT [32] or HAS-BLED [34] should be used to help quantify the risk of adverse effects and guide such decisions.
3. Cerebral microbleeds—the risk of bleeding must be weighed against the risk of stroke for the individual patient. Where patients have a low stroke risk, the risk of ICH with anticoagulants is likely to outweigh the benefits, but if the patient has a high stroke risk, then anticoagulation is beneficial.

## Conclusions

AF and VTE are common in older people as the risk increases with advancing age. Anticoagulants are an effective treatment for stroke prevention in AF and prevention of VTE recurrence, but they are often underused due to concerns about the adverse effects of falling whilst taking these medications. Whilst there is some evidence to suggest that falling whilst taking an anticoagulant may increase the risk of ICH and death, most of the work suggests that the absolute risk of these outcomes is low and outweighed by the reduction in stroke/VTE risk.

To minimise the risk associated with these medications, particularly in the case of frequent and recurrent falling, prescribers should conduct a multifactorial falls assessment and address modifiable risk factors such as concomitant medication which may no longer be required, mobility or balance issues and home hazards. When starting an anticoagulant for VTE, the duration of therapy should be clearly specified, and the ongoing risk reviewed at regular intervals. Off-label dose reduction of DOACs and lower INR targets have been used in an attempt to mitigate the bleeding risk associated with these agents; however, this is not recommended as it reduces their efficacy with little effect on bleeding risk. AF risk calculators should be used to help identify those at high risk of bleeding where anticoagulation should be avoided. Patient preferences and goals should inform decision-making particularly in frailer groups or in those approaching end of life where the benefits of anticoagulation may be attenuated.
